# Milk in Fevers

**Published:** 1886-01

**Authors:** R. W. White

**Affiliations:** Waco, Texas


### MILK IN FEVER.

*R. W. White, M. D., Waco, Texas.*

For Daniel's Texas Medical Journal.

HAVING noticed several articles lately on the subject of milk in fevers, I have concluded to give you my experience, as it extends over a period of thirty or more years.

I have found sweet milk inadmissible where the fever is high, while fresh buttermilk is not only grateful to the patient, but often sooths them and reduces the fever. Fresh buttermilk has been my principal diet in typhoid fever for over thirty years, and I do not now remember a solitary instance where it disagreed with the patient if they were not prejudiced against it in health.

